# Investigation of Förster Resonance Energy Transfer (FRET) and Competition of Fluorescent Dyes on DNA Microparticles

**DOI:** 10.3390/ijms16047738

**Published:** 2015-04-08

**Authors:** Jieun Kim, Jae Sung Lee, Jong Bum Lee

**Affiliations:** Department of Chemical Engineering, University of Seoul, Seoul 130-743, Korea; E-Mails: kjww9@uos.ac.kr (J.K.); jaesung88@uos.ac.kr (J.S.L.)

**Keywords:** DNA technology, DNA microparticles, bioimaging, Förster resonance energy transfer (FRET)

## Abstract

Fluorescent labeling is widely used to investigate the structural stability and changes to DNA nano- and microstructures. Despite this, the conventional method for observing DNA structures has several limitations in terms of cost-efficiency. This paper introduces a DNA spherical particle stained with DNA intercalating dyes (SYBR Green and SYTOX Orange) as tracers and reports the interaction between multiple dyes. The interference between the dyes was analyzed in terms of Förster resonance energy transfer (FRET) and competition. The changes in the fluorescence intensity by FRET were uniform, regardless of the sequence. The competition effect could occur when several dyes were added simultaneously. These properties are expected to help in the design of multicolor tracers in bioimaging and environmental applications.

## 1. Introduction

DNA has attracted considerable attention as a building block with unique self-assembly properties. A number of branched DNA molecules have been designed via a bottom-up assembly, such as two dimensional DNA crystals [1], DNA triangles and hexagonal tilings [2]. These branched structures have also been used to construct three-dimensional DNA architectures, such as DNA cubes [3], tetrahedrons [4], dendrimer-like DNA [5] and even macroscopic DNA hydrogels [6]. Moreover, using the rolling circle amplification method, spherical DNA structures independent of Watson-Crick base-pairing were introduced recently [7].

Their biological or medical applications are as varied as the many DNA structures because of their biocompatibility and biodegradability. For example, controlled drug delivery systems [8,9], switchable containers [10,11] and biosensing [12,13,14] have prompted intense investigation based on DNA. In addition, DNA has also been used for applications in environmental analysis [15,16] and biological imaging [17,18]. In this regard, the simultaneous monitoring of DNA particles is important for confirming the working of DNA particles, such as the cell uptake and detection.

To observe the DNA particles, quantum dots (QD) or fluorescent modified deoxynucleotides were normally utilized as a labelling agent [9,18,19]. On the other hand, they have some drawbacks, such as cytotoxicity [20] and low cost-efficiency, due to the complicated synthesis process [21,22]. Therefore, it is important to develop a tracer using DNA staining dyes and study the interactions between multiple dyes. In this study, two types of DNA spherical microparticles (ssDNA balls and dsDNA balls) were synthesized via RCA. In addition, the changes in fluorescence intensity based on Förster resonance energy transfer (FRET) and the competition between multiple DNA intercalating dyes were studied. By controlling the interaction between the DNA staining dyes, such as FRET and competition, DNA particles can be designed to emit a range of spectra that enable them to be used as powerful imaging tools in many applications.

## 2. Results and Discussion

### 2.1. Multicolored DNA Balls

**Figure 1:**
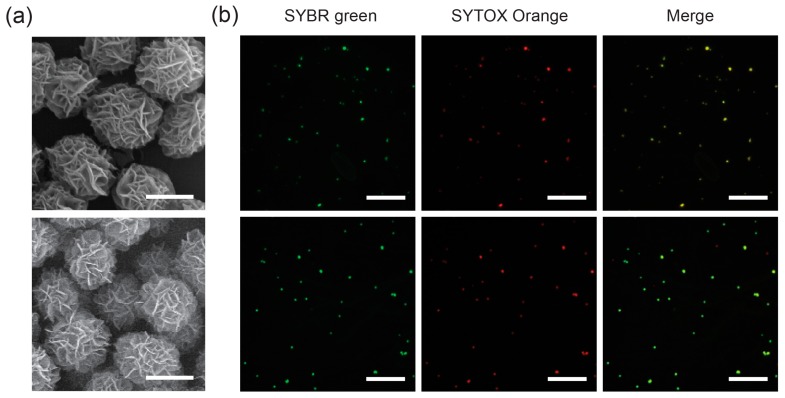
Scanning electron microscopy (SEM) and fluorescent microscopy images of ssDNA balls (**top** **panels**) and dsDNA balls (**bottom** **panels**). (**a**) SEM was used to analyze the morphology of the DNA balls; (**b**) Fluorescent microscopy images of the DNA balls stained with SYBR Green and SYTOX Orange. Scale bars, 1 μm in (**a**) and 10 μm in (**b**).

To examine the interference between dyes, two types of DNA balls, ssDNA balls and dsDNA balls, were first prepared by manipulating the template sequence. Scanning electron microscopy (SEM) was used to compare the morphology and structure of the DNA balls. The SEM images in Figure 1a indicate that they have a similar appearance, whose sizes are ~2 μm. Therefore, their externals will have little impact on the staining mechanism. Prior to quantitative analysis, it was confirmed that the DNA balls can be stained with multiple dyes. After staining each DNA ball with SYBR Green (SG) and SYTOX Orange (SO), fluorescence microscopy was used to visualize the DNA balls. The fluorescent microscopic images indicate that each ssDNA ball and dsDNA ball emits green and orange fluorescence simultaneously (Figure 1b).

**Figure 2:**
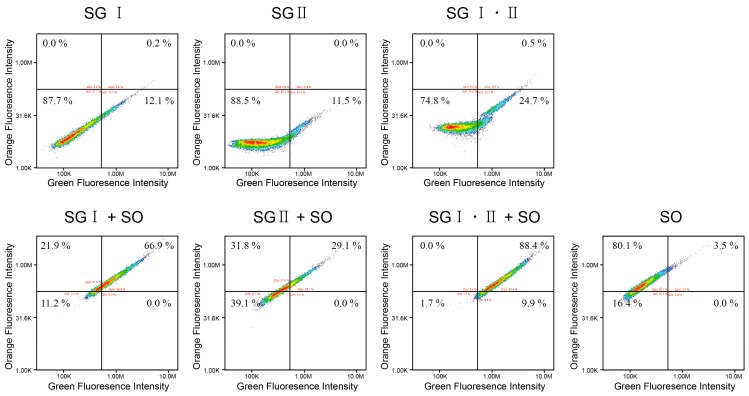
Image cytometry acquisition plots of two-color analysis of the ssDNA balls stained with SYBR Green or SYTOX Orange. The scatter plots show the green fluorescence intensity *versus* the orange fluorescence intensity. SG = SYBR Green; SO = SYTOX Orange.

Image cytometry was used to revalidate the staining of the DNA balls. The DNA balls stained with both green and orange dyes would show high-intensity on both green and orange. As shown in Figure 2, the ssDNA balls stained with SG I or SG II showed high values on the *x*-axis (more than 10% in the right lower quadrant), but showed low orange intensity. In contrast, only 3.5% of the balls were in the right upper quadrant and 80.1% were in the left upper quadrant when stained only with SYTOX Orange. These results suggest that the balls were stained well with the green and orange fluorescence dyes. The intensities of the ssDNA balls were shifted upward when the orange dyes were added.

**Figure 3:**
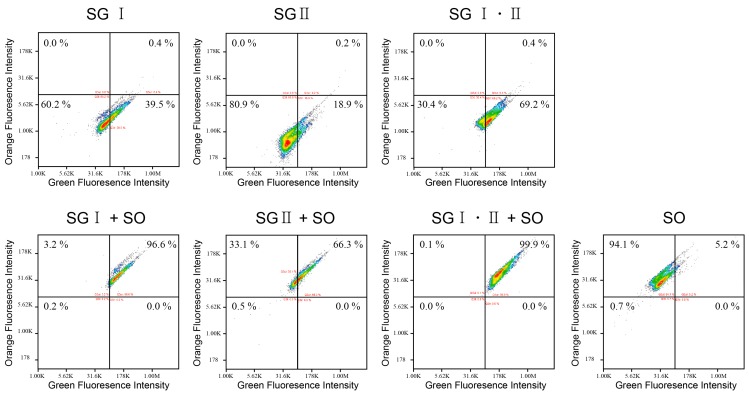
Image cytometry acquisition plots for two-color analysis of the dsDNA balls stained with SYBR Green or SYTOX Orange. The scatter plots show the green fluorescence intensity *versus* the orange fluorescence intensity. SG = SYBR Green; SO = SYTOX Orange.

Similarly, the dsDNA balls showed the same aspect in Figure 3. In the case of the dsDNA balls stained with SG only, strong green fluorescence intensities were measured (>18% in the right lower quadrant). While dsDNA balls that were stained only with SO showed strong orange fluorescence but rarely showed green fluorescence (percentage in the left upper quadrant and the right upper quadrant). The shifting of the intensity was also observed in the case of the dsDNA balls when the orange dyes were added. These results can be considered as the bases of the idea that DNA balls could be multicolored tracers.

### 2.2. Förster Resonance Energy Transfer (FRET) Efficiency Difference between ssDNA Balls and dsDNA Ball

**Figure 4:**
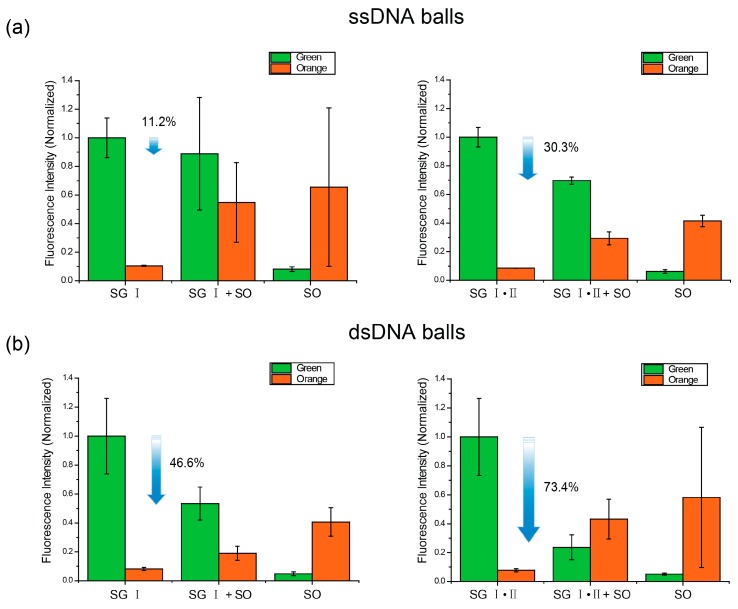
Intensity change in the DNA balls. Each balls were stained with either SYBR Green or SYTOX Orange or both. The blue arrow indicates the decrease in green fluorescence intensity. (**a**) The ssDNA balls were stained with the fluorescent dyes; and (**b**) The dsDNA balls were stained with the fluorescent dyes.

Because both SO and SG I are intercalating dyes, it can be expected that the degree of staining shows a difference between ssDNA balls and dsDNA balls. To compare the ssDNA balls and the dsDNA balls, the fluorescence intensities of the DNA balls stained with the DNA staining dyes were measured. In the case of the ssDNA balls stained with SG I, the green fluorescence intensity decreased by only 11.2% when the SO were added at the same time as SG I (Figure 4a, left). This suggests that there was little competition and energy transfer existed between SG I and SO because the DNA intercalating dyes bound to ssDNA through external binding, not intercalation [23]. Numerous strands of the ssDNA balls might have been sufficient to separate the dyes, an interaction between the dyes was not found. In addition, when the ssDNA balls were stained with SG I, II and SO, the green fluorescence intensity decreased by 30.3% from when they were stained only with SG I and II (Figure 4a, right). Compared to the previous result, the intensity decreased further because a number of single-strands in the ssDNA balls were stained with SG II. Therefore, the distance between the SG and SO decreased, which caused the FRET effect. In addition, in the case of the dsDNA balls, the decreases were generally larger than those of the ssDNA balls. As shown in Figure 4b left, the intensity decreased 46.6% because of SO. Both SG I and SO stain double-stranded DNA; Hence, they have to compete with each other. As a consequence of competition, SG I occupied little space. Furthermore, Figure 4b right revealed a considerable decrease in intensity (by 73.4%). Competitions on the sites for external binding between SG II and other dyes are negligible because there are sufficient external binding sites and the secondary dissociation constant of SO is relatively high [24]. Therefore, excess decline of the green fluorescence intensity can be explained by FRET. To confirm this notion, we performed FRET experiments and the resonance energy transfer between SG and SO was observed (see Figures S1).

### 2.3. Interference between the Dyes Dependent on Staining Method

**Figure 5:**
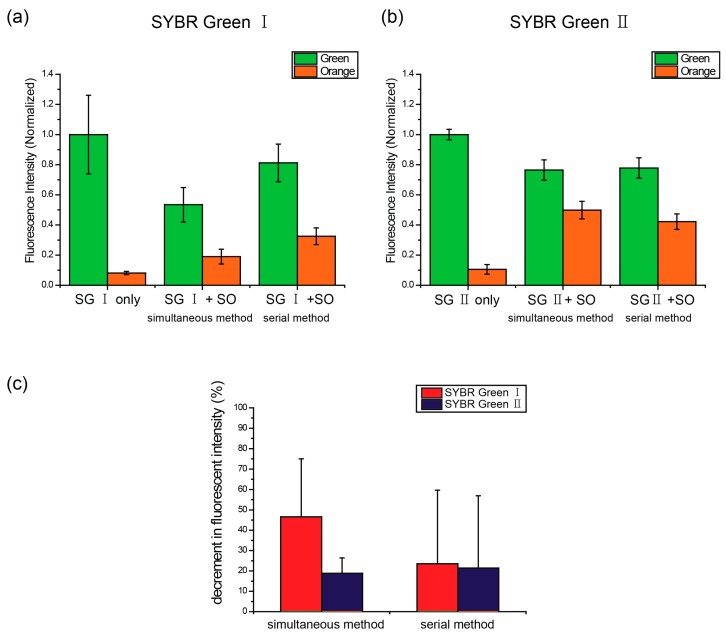
Alteration of the FRET dependent on the staining methods. The dsDNA balls were stained with the green and orange dyes simultaneously or in series. In the serial staining, SYTOX Orange was added after SYBR green staining (**a**) SYBR Green I was used as the green fluorescent dye; (**b**) SYBR Green II was used as the green fluorescent dye; and (**c**) Decrease in the green fluorescence intensity depending on the staining methods.

To analyze the competition more specifically, we stained dsDNA balls with the DNA staining dyes in series. After a sufficient staining time with SG I, SO was added. In the case of serial staining, the green fluorescence intensity decreased to less than that of simultaneous staining (Figure 5a,c). When the dsDNA balls were stained using the simultaneous method, the green fluorescence intensity decreased by 46.6%, whereas it decreased by 18.8% in the serial method. These results mean that the decrease in intensity was dependent on the staining method. The serial method enabled the green fluorescent dye to intercalate sufficiently so they did not have to compete with each other. Therefore, it can be considered that a decrease in the serial method was entirely due to FRET. This was also confirmed by the experimental results, as shown in Figure 5b. Each dsDNA ball was stained with SG II and SO using simultaneous and serial methods. As shown in Figure 5b,c, both methods resulted in a decrease in green fluorescence intensity; however, there were few gaps between them (23.5% decrease in the concurrent method and 22.2% decrease in the serial method). In addition, the decreases were similar to the results of serial staining with SG I (Figure 5a,c). Therefore, these results support the idea that the decline in intensity with the serial method was caused by FRET.

## 3. Experimental Section

### 3.1. Preparation of Circular DNA

To synthesize the circular DNA, two different phosphorylated linear ssDNA were designed rationally. Linear DNA 1 was designed to form hairpin structures and linear DNA 2 was designed to be composed of single-stranded DNA. The DNA sequence of the linear strands is as follows: linear DNA 1 is 5'-Phosphate-ATA GTG AGT CGT ATT AAC GTA CCA ACA AAT GTG AAT GCA GAC CAA AGA ATT ACT TGA ATT CTT TGG TCT GCA TTC ACA TTT TAG AGG CAT ATC CCT-3', and the sequence of primer 1 (a sequence complementary to the linear DNA) is as follows: 5'-TAA TAC GAC TCA CTA TAG GGA T-3'. Linear DNA 2 is 5'-Phosphate-ATA GTG AGT CGT ATT AAC GTA CCA ACG TTC GAT CGC GAG AAA ATT CGA ATC ATC CCC AAT AGA GGC ATA TCC GCA TAT AAG ATG CGA CCG CT-3'. In addition, the sequence of primer 2 is 5'-ACT CAC TAT AGC GGT CGC AT-3'.

Each linear DNA was mixed with their primer in nuclease-free water. For denaturation and annealing, each mixed solution was heated to 95 °C for 2 min and cooled gradually to 25 °C for 1 h. To connect the nick of the circular DNA, each solution was incubated overnight at room temperature with T4 DNA ligase (3 U/μL) and ligase buffer (300 mM Tris-HCl (pH 7.8), 100 mM MgCl_2_, 100 mM dithiothreitol and 10 mM adenosine triphosphate).

### 3.2. Synthesis of DNA Balls

To synthesize ssDNA balls and dsDNA balls, each circular DNAs prepared in the previous step (3 μM) were mixed with Φ29 DNA polymerase (100 U/μL), deoxyribonucleotide triphosphate (2 mM) and reaction buffer (40 mM Tris-HCl (pH 7.5), 50 mM KCl, 10 mM MgCl_2_, 5 mM (NH_4_)_2_SO_4_ and 4 mM dithiothreitol). For the RCA process, each solution was incubated for 20 h at 30 °C. To refine and collect the DNA balls, the resulting products were sonicated and washed several times using nuclease-free water.

### 3.3. Multicolor Staining of the DNA Balls

For energy transfer, SYBR green I, II and SYTOX Orange were selected. The DNA-SYBR green-complex absorbs blue light (497 nm) and has a maximum of emission at 520 nm (see Figure S2). SYTOX Orange has an absorption maximum at 547 nm and emits orange light (570 nm) (Figure S2). Because their excitation and emission wavelength range are overlapped and both are DNA intercalating dyes, they can be a donor and accepter (see Figure S3; The Förster distance in supplementary information page 5).

For microscopy analysis, each ssDNA balls and dsDNA balls were stained with SYBR Green (10^−4^ dilution of the stock solution, Invitrogen, Carlsbad, CA, USA) and SYTOX Orange (2.5 × 10^−2^ mM final concentration, Life Technologies, Carlsbad, CA, USA) for 6 h. The green fluorescent stained DNA balls and the orange fluorescent stained DNA balls were observed by fluorescent microscopy (Nikon, Tokyo, Japan, Eclipse Ti). SEM (Hitachi, Tokyo, Japan, S-4200) was used to compare the morphology of the ssDNA balls and the dsDNA balls. The samples were dried on silicon wafers. Image cytometry was performed using a Nucleo Counter (Chemometec, Copenhagen, Denmark, NC-3000) to confirm the capacity of the DNA balls (Green: λ_ex_ = 475 nm & λ_em_ = 560 nm, Orange: λ_ex_ = 530 nm & λ_em_ = 675 nm). For a sufficient staining, the DNA balls were incubated with the staining dyes overnight.

### 3.4. Measurement of Fluorescence Intensity

To confirm the FRET effect dependent on the sequence of the DNA balls, each ssDNA balls and dsDNA balls were stained separately with SYBR Green I (10^−4^ dilution of the stock solution, Invitrogen) and SYTOX Orange (2.5 × 10^−2^ mM final conc., Life Technologies) for 6 h. After staining the DNA balls, green and orange fluorescence (λ_ex_ = 485 nm & λ_em_ = 528 nm, λ_ex_ = 530 nm & λ_em_ = 590 nm) was measured using a fluorescence microplate reader (BioTek, Winooski, VT, USA, Synergy HT). Dilute staining dyes (same concentration as above) were used as blank solution to remove background.

Two different staining methods were used to observe the change in the fluorescent decreasing ratio dependent on the competition. For simultaneous staining, the DNA balls were stained simultaneously with both SYBR Green and SYTOX Orange overnight. In the sequential method, each DNA balls were stained with SYBR Green I or SYBR Green II for 6 h first. SYTOX Orange was then added to the previous solutions and incubated overnight. The fluorescence (λ_ex_ = 485 nm & λ_em_ = 528 nm, λ_ex_ = 530 nm & λ_em_ = 590 nm) was measured using a fluorescence microplate reader (BioTek).

## 4. Conclusions

DNA balls can be stained with multiple dyes and their fluorescence intensities are influenced by several factors. The decrease in fluorescence intensity depends on the sequence of the DNA templates, the type of staining dye and the staining steps. The decrease in the fluorescence intensity by Förster resonance energy transfer in the DNA balls was measured using both the intercalating dye and ssDNA binding dye. The results show that there are 20% to 30% of intensity changes because of FRET. In addition, the competition of intercalating dyes was observed in the existence of double-stranded DNA, when multiple dsDNA-specific dyes were added to the solution simultaneously. On the other hand, we did not see any significant decrease of fluorescence intensity by adding DNA specific dyes in serial method, suggesting there was less competition between the dyes. Therefore, to increase the binding ratio of DNA specific dyes to DNA ball containing a large amount of double-stranded DNA, the desired dye should be added first and be incubated for a sufficient time. From these results, DNA balls can be designed to fluoresce various colors as multicolor tracers in bioimaging and environmental applications.

## Supplementary Materials

Supplementary materials can be found at http://www.mdpi.com/1422-0067/16/04/7738/s1.
