# Supplementary material for: Investigation of Förster Resonance Energy Transfer (FRET) and Competition of Fluorescent Dyes on DNA Microparticles
Source: Int J Mol Sci. 2015 Apr 8;16(4):7738–47. doi: 10.3390/ijms16047738 (PMC4425046; doi:10.3390/ijms16047738)
Supplement: Supplementary file 1 [file ijms-16-07738-s001.pdf]

Supplementary Information

Resonance Energy Transfer Between SG and SO

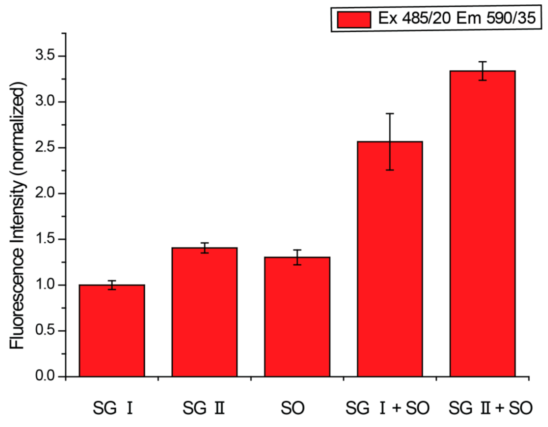

Figure S1. Fluorescence intensity of dsDNA balls which measured using Plate Reader (λ_{ex} = 485 nm, λ_{em} = 590 nm). dsDNA balls were stained with SYBR Green (10^{-4} dilution of the stock solution, Invitrogen) and SYTOX Orange (2.5×10^{-2} mM final conc., Life Technologies) for 6 h.

The results showed that the fluorescence intensities are increased when the dsDNA balls were stained with both SG and SO compared to the case of staining with SG or SO only. In addition, the intensity of the SG-SO double stained dsDNA balls were larger than sum of the SG stained dsDNA balls and SO stained dsDNA balls. The fluorescence intensities of SG I—SO pair and SG II—SO pair were increased by 11% and 23% in each case, confirming FRET between the dyes.

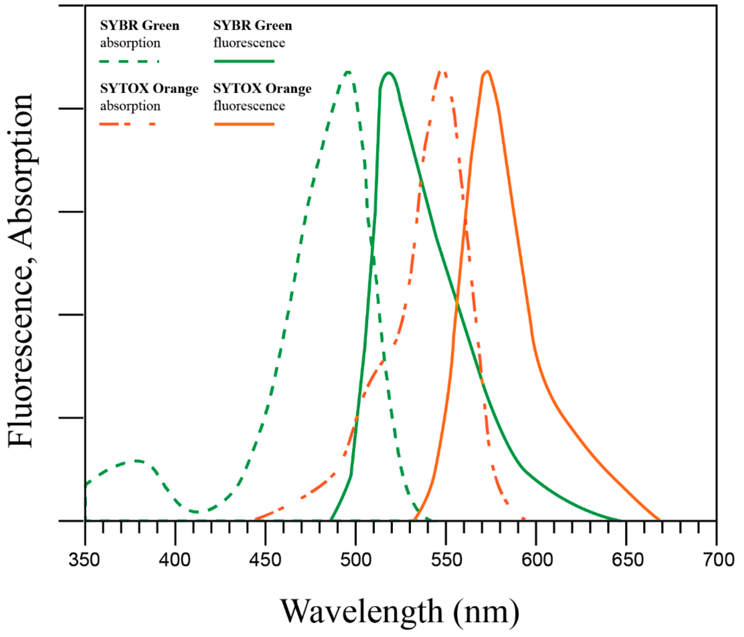

Figure S2. Excitation and emission spectra of SYBR green and SYTOX Orange. Normalized absorption spectra of SYBR Green (green dash lines) and fluorescence spectra of SYBR Green (green solid lines). Normalized absorption spectra of SYTOX Orange (orange dash lines) and fluorescence spectra of SYTOX Orange (orange solid lines). The data of SYBR Green and SYTOX Orange were from Life Technologies.

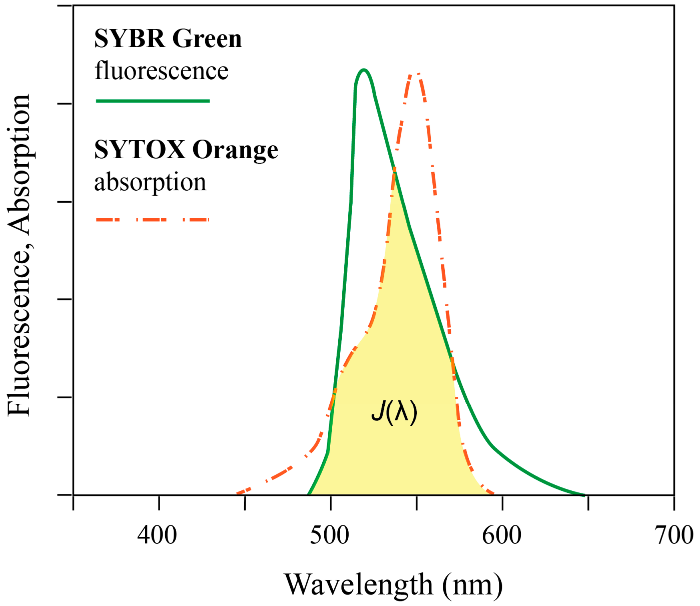

Figure S3. Excitation and emission spectral overlap. Normalized absorption spectra of SYTOX Orange (orange dashed lines) and fluorescence spectra of SYBR Green (green solid lines). The data of SYBR Green were from Invitrogen and the data of SYTOX Orange were from Life Technologies.

Computation of SG-SO Förster Distance

The Förster distance (R_0), the distance at which the energy transfer efficiency is 50%, was calculated by following Equation [25]:

$$R_o = 0.2108[k^2 Q_D n^{-4} J(\lambda)]^{\frac{1}{6}}, \qquad \text{Å} \tag{1}$$

$$J = \int f_D(\lambda)\varepsilon_A(\lambda)\lambda^4 d\lambda \tag{2}$$

where $k^2 = 2/3$ is the dipole orientation factor, assuming the free rotation of the dyes. $Q_D = 0.8$ is the fluorescence quantum yield of SYBR Green [26] and $n = 1.33$ is the refractive index of the media (water). J is the spectral overlap integral, where $f_D(\lambda)$ is the normalized emission spectra of SYBR Green and $\varepsilon_A = 79{,}000\ M^{-1}\cdot cm^{-1}$ is the molar extinction coefficient of SYTOX Orange [27]. The calculated Förster distance of SG-SO pair is $R_0 = 59.2$ Å.
